# Capture myopathy in hooved mammals and human Takotsubo syndrome

**DOI:** 10.1093/emph/eov023

**Published:** 2015-09-28

**Authors:** John E. Madias

**Affiliations:** Icahn School of Medicine at Mount Sinai, New York, NY, and the Division of Cardiology, Elmhurst Hospital Center, Elmhurst, NY, USA

To the Editor

I read with great interest the study by Blumstein *et al.* [1], published online ahead of print on 21 July 2015 in the *Journal*, about capture myopathy (CM) and its clinical implications for human Takotsubo syndrome (TTS), which reminded me of the seminal work done by Ueyama *et al..* [2] with their rat immobilization animal model of TTS. The capacity of the brain to injure the heart is well established, based on clinical studies and animal experimentation for over a century [3]. The authors of this study hypothesize that ‘CM syndromes in wildlife may be a model for human stress cardiomyopathy, including TTS’, which is very plausible, and in this respect it may be advisable for the workers in the field of human TTS to explore in patients some traits that they have detected in animal CM [1]. Since the authors found ‘greater brain mass, faster maximum running speed, greater minimum group size, and greater maximum longevity’, in animal susceptible to CM, it may be contributory to evaluate in patients with TTS, the size of their brain, based on imaging studies, their history of running speed, and their sociality. Regarding the longevity trait there is similarity in animals with CM and patients with TTS, since the latter mainly strikes the elderly humans, mainly women [4]. Patients with history of depression, anxiety, and posttraumatic stress disorder are susceptible to TTS, which is a parallel to the authors’ remarks about species ‘that have successfully avoided predation’, and which seem to be more likely to be susceptible to CM [1]. The authors appear to expect progress in their field by more systematic and thorough necropsy of carcasses of victims of CM [1], although they also maintain that ‘CM is probably a continuum of effects, possibly affecting a majority, or even all animals that are captured, with an unknown portion of those affected to the point of showing signs recognizable as CM’. This fits with this author’s beliefs, that there exist milder atypical forms of human TTS [5]. Since we do not have access to symptoms, but only to signs, or laboratory expressions of the disease in afflicted animals with CM, it behooves workers in this field to employ measurements of blood catecholamines, creatine kinase and troponins, and systematic implementation of electrocardiography for detection of transient changes (ST-segment elevation and depression, T-wave inversion, and QT interval prolongation), and echocardiography-based reversible myocardial wall motion abnormalities.

**Conflict of interest**: None declared.
